# A new approach for analysis of heart rate variability and QT variability in long-term ECG recording

**DOI:** 10.1186/s12938-018-0490-8

**Published:** 2018-05-03

**Authors:** Hau-Tieng Wu, Elsayed Z. Soliman

**Affiliations:** 1 Department of Mathematics and Department of Statistical Science, Duke University, 207 Physics Building, 120 Science Dr, Durham, NC 27705 USA; 2 Mathematics Division, National Center for Theoretical Sciences, Taipei, Taiwan; 3 Epidemiological Cardiology Research Center (EPICARE), Department of Epidemiology, Wake Forest School of Medicine, Winston-Salem, NC USA; 4 Department of Internal Medicine, Section on Cardiology, Wake Forest School of Medicine, Winston-Salem, NC USA

**Keywords:** Heart rate variability, QT variability, Long-term ECG recording, Time–frequency analysis, Concentration of frequency and time (ConceFT)

## Abstract

**Background and purpose:**

With the emergence of long-term electrocardiogram (ECG) recordings that extend several days beyond the typical 24–48 h, the development of new tools to measure heart rate variability (HRV) and QT variability is needed to utilize the full potential of such extra-long-term ECG recordings.

**Methods:**

In this report, we propose a new nonlinear time–frequency analysis approach, the concentration of frequency and time (ConceFT), to study the HRV QT variability from extra-long-term ECG recordings. This approach is a generalization of Short Time Fourier Transform and Continuous Wavelet Transform approaches.

**Results:**

As proof of concept, we used 14-day ECG recordings to show that the ConceFT provides a sharpened and stabilized spectrogram by taking the phase information of the time series and the multitaper technique into account.

**Conclusion:**

The ConceFT has the potential to provide a sharpened and stabilized spectrogram for the heart rate variability and QT variability in 14-day ECG recordings.

**Electronic supplementary material:**

The online version of this article (10.1186/s12938-018-0490-8) contains supplementary material, which is available to authorized users.

## Background

The non-stationary dynamic nature of the heart rate renders the heart rate variability (HRV) analysis, and subsequently the RR interval time series analysis and QT interval time series analysis, a difficult task. Nevertheless, several methods are currently used to quantify the HRV from 24 to 48 h long-term electrocardiogram (ECG) recordings [1–5]. These methods could be briefly classified into four major categories—time domain approach, frequency domain approach, nonlinear geometric approach, and information theory based approach [6–9]. Most of the methods applied to measure long-term HRV are based on the stationarity assumption [1–3, 10], a common assumption in many time series techniques. While those methods could still be applied to any non-stationary time series, such as 24–48 h long-term heart rate, the results might not be directly interpretable, miss the finer non-stationary dynamics, or even misleading [11]. The problem becomes more challenging with “extra-long-term” ECG recordings, such as 7 days or longer [4, 12].

To handle the non-stationarity challenge, it is possible to truncate the time series into overlapping or non-overlapping segments, and then evaluate the spectral content. If the assumption that the time series are locally stationary without frequent dramatic changes holds, such an approach could capture the dynamic information. This approach is called the Time Frequency (TF) analysis [13]. Short Time Fourier Transform (STFT) [14] and Continuous Wavelet Transform (CWT) [15] are common linear-type TF analysis tools, and Cohn’s classes [16] are common quadratic-type TF analysis tools (smoothed pseudo-Wigner-Ville transform (SPWVT) [17] and Choi-Williams distribution (CWD) [18] are common choices of Cohn’s classes). These algorithms have been widely applied to HRV measurements [14–21]. Since the instantaneous spectral content is the main information to extract, the TF analysis could be classified as the *frequency domain* method. While these algorithms have been widely applied, they are not free of limitations. The main limitation of linear-type TF analysis techniques such as STFT and CWT is the blurring effect caused by the uncertainty principle [22]. This blurring effect might mask the available information and downgrade the analysis quality [23]. The quadratic-type TF analysis tools generally lose the causality information, and possible interference patterns might lead to an artificial outcome.

In this report, we focus on the TF analysis approach and propose a new nonlinear-type TF analysis approach, the concentration of frequency and time (ConceFT) [24], to study HRV and QT variability from extra-long-term ECG recordings. ConceFT is a generalization of the above-mentioned TF analysis techniques like STFT or CWT. It resolves the blurring effect limitation commonly encountered in the linear-type TF analysis techniques by sharpening instantaneous spectral content. It does this by taking the phase information of the time series and the multitaper technique into account, and by being stable to noise. The ConceFT has been previously shown to be an ideal tool to capture the dynamic spectral content of the RR interval time series that much better quantify anesthetic depth and noxious stimulation during the surgery under general anesthesia [23, 25]. Also, the idea of ConceFT was successfully applied to quantify the variability of the electrical activity of the diaphragm and the respiratory pressure signal to show how the mechanical ventilation impacts the breathing variability in infants [21].

## Methods

As proof of concept of our approach, we utilized data from four long-term ECG recordings, about 2 weeks (about 325 h) each. The data was recorded using ZIO^®^ Patch (iRhythm Technologies, Inc., San Francisco, California, USA) with a sampling rate of 200 Hz. The underlying information of the subjects was unknown to us.

### Concentration of frequency and time–time-varying power spectrum

To take into account the non-stationarity of a given time series, such as the physiological non-stationarity status of the HRV time series, and to avoid the limitations encountered in linear-type and quadratic-type TF analysis tools, we considered the recently developed nonlinear-type TF analysis technique ConceFT. ConceFT has the ability to sharpen the spectrum, capture the non-stationary dynamics, and possesses stability in regard to noise [23]. ConceFT is a generalization of the widely-applied multitaper (MT) technique [26] to the nonlinear-type TF analysis. Here we delineate the needs for ConceFT and summarize the algorithm step by step.

As mentioned, the basic idea of TF analysis is truncating the given time series by a given window at a different time, evaluating the power spectra of those truncated segments, and then stitching the power spectra obtained at those different times to create a spectrogram [13]. Notably, the multiplication of any window with the time series at time *t* means that the analysis on the time series is focused around time *t*. The first limitation of this approach is the broadening or smearing of the spectral content caused by the uncertainty principle [22]. When noise exists, it is natural to ask if the algorithm is stable to noise. The MT technique [26] is widely applied to stabilize the algorithm. When applying the MT technique, the TF representations obtained from multiple orthonormal windows are averaged. While the MT technique could help obtain stable information about the non-stationary dynamics, this approach is limited by the “Nyquist rate”; that is, the number of windows competing with the desired TF resolution [27].

To alleviate the first limitation, the spectrum could be sharpened by taking the phase of the Fourier transform of the truncated segment into account. This nonlinear approach is called synchrosqueezing transform (SST) [28]. For the second limitation, the nonlinear nature of the sharpening procedure could be taken into account to generalize the MT technique by considering multiple linear combinations of the chosen orthonormal windows. This combination leads to ConceFT, which binds the spirit of the MT technique and the nonlinearity of a chosen TF analysis.

ConceFT is composed of three steps. The first is choosing *J* orthonormal windows, where *J* is a positive integer, and for that we choose the first *J* Hermit windows due to their optimal time–frequency concentration [27]. In order to capture the local dynamics of the time series, we take *N* linear combinations of those *J* orthonormal windows so that the new window is of unit norm. Second, we evaluate the STFT by multiplying each linearly-combined window to the time series centered at a different time, and evaluating the Fourier transform. For each linearly-combined window and each time, we take the phase information of the Fourier transform to sharpen the spectral content; that is, the SST is applied [28]. Finally, at each time, we average the sharpened spectral contents evaluated by *N* different linearly combined windows, and obtain the final result, which is again a spectral content. By stitching the sharpened and stabilized power spectra obtained at a different time, we obtain a new spectrogram, which we call the *time*-*varying power spectrum* (tvPS). The result is a sharpened and stabilized spectrogram [23].

### How to read the time-varying power spectrum

**Fig. 1:**
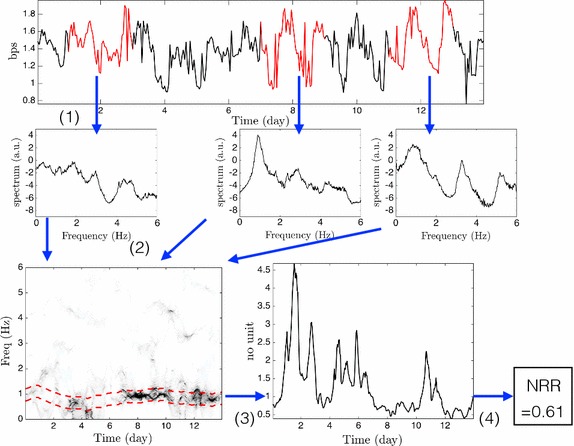
The flowchart of the proposed approach to study the long-term recording HRV. The time series shown in the top is constructed by the RR interval via the interpolation, and smoothed for the visualization purpose. (1) The truncated segments of the heart rate time series are marked in red. In this step, we apply the concentration of frequency and time, which is composed of the synchrosqueezing transform and the generalized multitaper algorithm, to estimate the power spectrum associated with different segment. To enhance the visualization, the power spectrum is plotted in the log2 scale. (2) All estimated power spectra are stitched together according to time, which ends up with an image (time–frequency representation) called the time-varying power spectrum generated by ConceFT. (3) Determine the dominant curve in the tvPS and evaluate the energy around the dominant curve at each time. Then evaluate the ratio of the energy concentrated on the band marked by the red dashed curves and the remaining energy. As a result, we obtain the non-rhythmic to rhythmic ratio (NRR) function. (4) The standard deviation of the NRR function is the summary index called the NRR index. This index quantifies how concentrated the tvPS is around the dominant spectral component

The tvPS is represented as a matrix, which is usually represented as an image for visualization. See Fig. 1 for a typical tvPS generated by ConceFT with the 14-day heart rate signal. The *x*-axis in Fig. 1 indicates the time in days. The *y*-axis indicates the frequency with in “Hz.” As a TF representation, this image comes from stitching together sequential spectra indexed by time. At each slice indexed by time, the intensity on that slice indicates how strongly the signal oscillates at the frequency indexed by the frequency axis. According to the developed theory [23, 28], the main information we can acquire from the tvPS is the “curve” pattern. Particularly, an oscillatory component inside the time series is represented as a curve indexed by time. In this example, after day 7, there is a dominant oscillatory component at 1 Hz, which indicates a daily oscillation inside the heart rate signal. Theoretically, this curve indicates the time-varying frequency of the oscillatory component, and the intensity of the curve indicates the strength of that component [23].

### Summarize the tvPS information—long-term variability index

While ConceFT provides stabilized and sharpened dynamic spectral information by the tvPS, for the clinical application, we may prefer a more simplified index that could faithfully summarize the information provided by the tvPS. There are several different ways to achieve this goal. One approach is taking the distribution pattern of the tvPS into account, since it provides different dynamic physiological information that depends on the physiological background. To quantify the distribution pattern, we first obtain a *time*-*varying parameter* over a predesigned spectral region, like the time-varying low-frequency power [25], or describe how regularly the signal is oscillating, like the rhythmic to non-rhythmic ratio (NRR) [21]. These time-varying parameters decouple specific information from the complicated integrated information in the heart rate. For example, the time-varying low-frequency power captures the sympathetic tone information, and the NRR captures the respiratory sinus arrhythmia. Next, we can consider a model approach to estimate the underlying parameters associated with the time-varying parameter, or summarize the dynamics by imposing stationarity [6–9]. We illustrate the whole procedure in Fig. 1 with the 14-day heart rate signal.

### Data preprocessing

To apply ConceFT to analyze the HRV, we carry out the following ECG preprocessing steps: R-peak and T-end detection, correct RR interval to adjust the error incurred by artifacts or arrhythmic beats, and correct QT interval. If the ECG tracking is sampled at a frequency lower than 500 Hz, we follow the suggestion of the Task Force [29] and [30] and upsample the ECG tracking to 500 Hz. The power line interference is filtered using a notch filter. The baseline wandering is filtered using a median filter with the window length 500 ms long.

### R peaks detection

The R-peaks are detected by the QRS detection algorithm [31]. A refractory period of 250 ms is considered to remove obvious artifacts [32]—when two peaks are detected with the period shorter than 250 ms, the peak with the sharpest slope is retained. To further remove the artifacts incurred by the spurious R-peak detection or missing R-wave detection, we apply the median filter of five consecutive RR intervals, which is a robust estimate of the expected RR interval. The RR interval that dramatically deviates from this median is labeled as a suspected RR interval. If a suspected RR interval is significantly shorter than the median (we used 0.5 times as the criterion in this analysis), and the sum of two adjacent RR intervals is close to the median, this suspected RR interval is considered coming from spurious R-peak. In this case, the spurious R-peak is removed. If the suspected RR interval is significantly longer than the median (we used 2 times as the criterion in this analysis), it is possible that one or more R-peaks are missed. In this case, the interval is divided into *k* segments, where *k* is the rounding integer of the division of the suspected RR interval and the median.

### Arrhythmic beats editing

It is well known that arrhythmic beats could introduce a bias into HRV analysis. For example, ectopic beats impair the reliability of the different approaches for the RR interval time series analysis, like artificially increasing the high frequency band power in the spectral method and the standard deviation in the time domain method. In our analysis, we distinguished sinus beats from abnormal beats by the currently proposed classifier for the single lead ECG signal [32]. We combined the beat-to-beat statistics and the time domain features, and applied the support vector machine (SVM) [33] to distinguish sinus rhythmic beats from the abnormal beats. Particularly, we considered the following features: R-peak amplitude, the time difference between current and previous beat (at R-peak), the time difference between current and next beat (at R-peak), the average R-peak to R-peak interval over 10 beats, the phase associated with the R-peak, and the duration of the QRS complex. These features are chosen since they are simple to acquire and relatively robust in the presence of noise. The SVM is trained from the MIT-BIH arrhythmia database [34], which contains 48 half-hour two-lead ambulatory ECG signals (denoted as lead A and lead B). We followed the ANSI/AAMI EC57:1998 standard [35] and excluded paced records. The training is carried out on the lead II signals of the remaining cases.

After finding those arrhythmic beats, we followed the suggested procedure [36] to edit the RRI associated with the arrhythmic beats and the following compensatory pause beat. First, we excluded 10-min segments that have less than 80% normal beats [36]. For the other segments, we used the non-linear predictive interpolation for RRI artifact correction [37]. We used the consecutive 10 RR intervals before the abnormal RR interval and then find the consecutive 10 normal RR intervals that are closest to the particular segment with the abnormal RR interval. The RR interval following the chosen consecutive 10 normal RR intervals was used to replace the RR interval for the abnormal beat. More details on the non-linear predictive interpolation are available somewhere else [8, 38, 39].

### QTc evaluation

The beginning and the end of the QRS complex, along with the maxima of the complex are detected, after correcting the polarity as needed. The end of T-wave position is determined by the area measuring method [40]. With the timestamp of the end of each T-wave, we obtained the QT interval.

Individual variations in the relationship between QT and RR intervals are well established [41], and therefore the heart rate-corrected QT interval (QTc) is better evaluated using the individual-specific corrections by the linear regression techniques [42] as follows: Denote *RR*(*i*) to be the corrected *i*-th RR interval, measured in seconds. Denote *QT*(*i*) to be the length of the QT interval associated with the *i*-th ventricular response. Then, correct the *i*-th QT interval by locally fitting the *41* closest RR intervals by the formula *QT* = *βRR*^*α*^, where *α* and *β* are constants, by the linear regression. The *i*-th QT interval is corrected by the associated *α* via $QT_{c} (i) = \frac{QT(i)}{{RR(i)^{\alpha } }}$ [42].

### Time series to be analyzed

To analyze the corrected RRI time series, the following time series were considered: 0 ≤ *γ* ≤ 1, and defined a time series *R*_*hour*,*γ*_ as *R*_*hour*,*γ*_(*i*) = *γ* quartile of RRIs in the *i*-th hour. Note that when *γ* = 1, we evaluated the maximal. In this study, we illustrate how the proposed ConceFT is able to analyze *R*_*hour*,0.01_, *R*_*hour*,0.99_ and *R*_*hour*,0.5_, which are all of about 325 in length since the recorded signals are about 325 h long. We view *R*_*hour*,0.01_ and *R*_*hour*,0.99_ as surrogates of measuring the minimal and maximal heart rate sampled uniformly each hour. These surrogates are chosen to avoid the possible outliers which may exist even after the correction.

It is well known that the variability of the beat-to-beat QT time series measures the stability of the ventricular repolarization duration, if we assume that the depolarization is more stable compared with the repolarization duration [30, 43]. As it provides physiological information from a different angle compared with the RR interval time series, we also consider the QTc time series to demonstrate how the ConceFT could be applied. For the variability analysis of the QT time series, we define the time series *Q*_*hour*,0.01_, *Q*_*hour*,0.99_ and *Q*_*hour*,0.5_ for the QTc.

We could also define other time series—for example, the one sampled per minute, if needed. However, to keep the illustration concise, we only focus on the above-mentioned time series associated with the RR interval and QT interval time series.

## Results

### Simulated signal

**Fig. 2:**
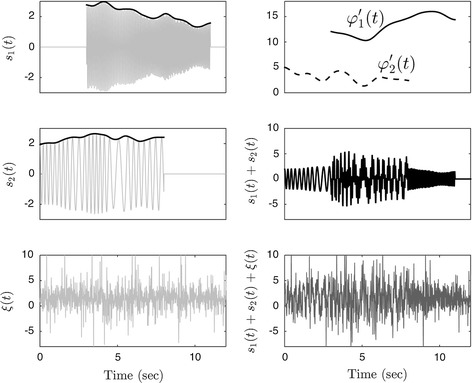
The simulated signal. The signal, shown on the right bottom subplot, is composed of two oscillatory components *s*_1_(*t*) and *s*_2_(*t*), and the noise *ξ*(*t*), shown on the left column. The time-varying amplitudes are superimposed on the left top and left middle subplots in black, and the time-varying frequencies are plotted on the right top subplot. It is clear that the amplitude and frequency are time-varying, and each component exists only for a finite period. The noise is spiky due to the fat-tail property of the considered noise

**Fig. 3:**
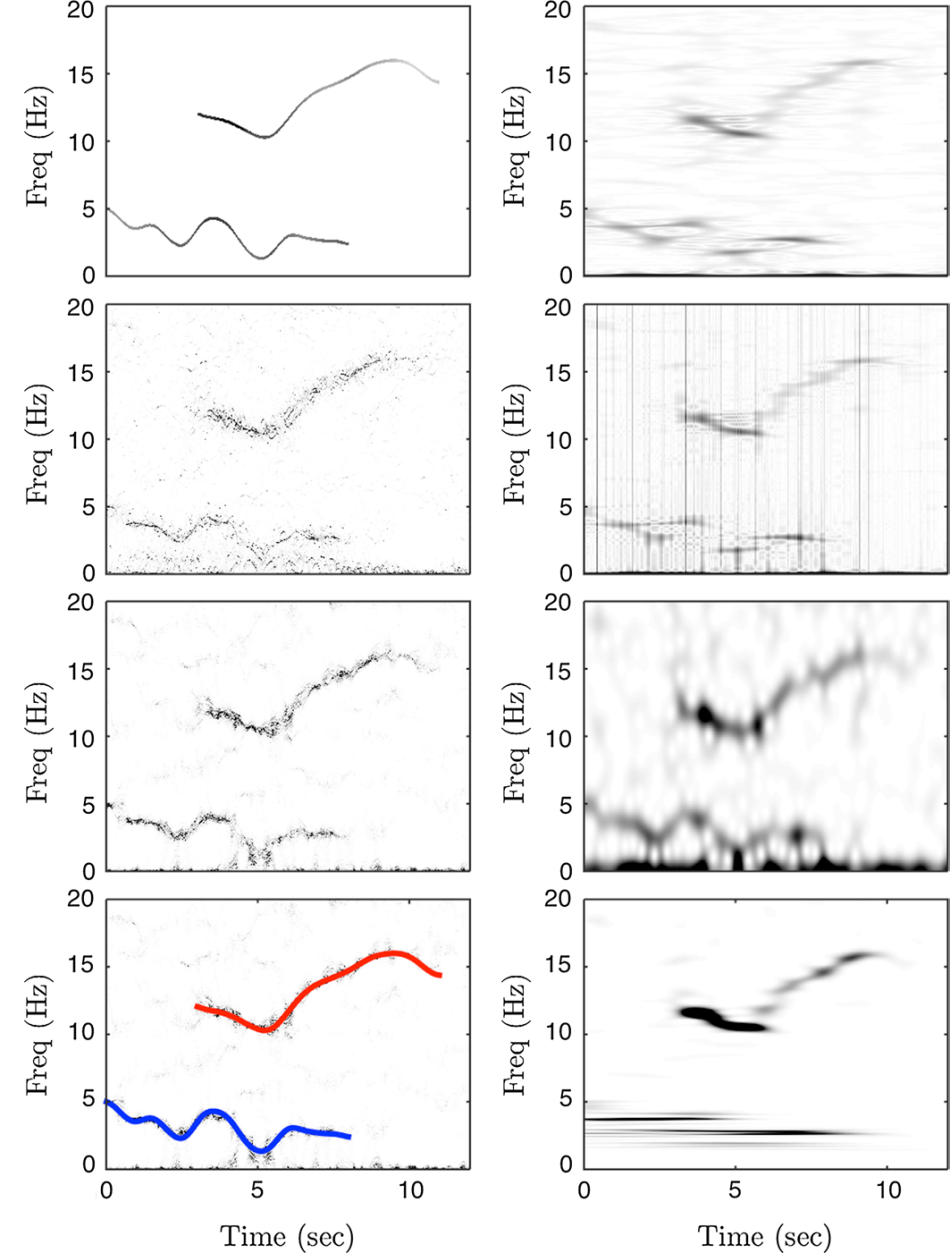
A comparison of different time–frequency (TF) analysis tools on the simulated signal. On the left column, from top to bottom, we show the ideal TF representation, the multitaper (MT) synchrosqueezed spectrogram, the concentration of frequency and time (ConceFT), and the ConceFT superimposed with the ground truth time-varying frequencies of the two components. On the right column, from top to bottom, we show the smoothed pseudo Wigner-Ville distribution (SPWVD), the Choi-William distribution (CWD), the spectrogram, and the scalogram. The ideal TF representation is the ground truth that we would like to recover. It encodes the precise frequency and amplitude of the oscillatory components. Interested reader could read Eq. 4.2 in (1) for details. It is clear that while the dynamics (like time-varying frequency and existence period) can be captured, ConceFT provides a sharper result compared with other approaches

To illustrate the performance of ConceFT, we demonstrated how it works in a simulated multi-component signal, and compared the resulting tvPS with those generated by the commonly applied TF analysis tools, like MT synchrosqueezed spectrogram, spectrogram (the square modulus of STFT), scalogram (the square modulus of CWT), SPWVD, and CWD. We modeled the signal to have two oscillatory components, each with a time-varying amplitude and frequency, and modeled the time-varying amplitudes and frequencies by realizations of smoothed Brownian paths [23]. We chose the smoothed Brownian path for our model since it cannot be represented by well-known functions [23] and hence it provides a more realistic model. For each component, we modeled it to exist only for a finite period which modeled the unexpected exterior stimulation that might change the dynamics. We then modeled the stochasticity by the autoregressive and moving average (ARMA) process. We considered an ARMA(1, 1) model determined by an autoregression polynomial *a(z) *= *0.5z *+ *1* and a moving averaging polynomial *b(z) *= − *0.5z *+ *1*; for the innovation process we used independent and identically distributed Student t4 random variables. We chose this ARMA(1, 1) random process since its time-dependent property better captures the time-dependent physiological property. The Student t4 random variable could well model the noise generated by “spurious beats” or “missing beats” since it has a fat-tailed distribution. See Fig. 2 for one example of the selected simulated signal. The signal is sampled at 100 Hz, and the signal-to-noise ratio is − 2 dB. We then apply ConceFT, MT synchrosqueezed spectrogram, spectrogram, scalogram, SPWVD, and CWD to the simulated signal. In the MT synchrosqueezed spectrogram, we followed the same parameters suggested by Daubechies et al. [23] and chose the first 6 Hermit polynomials with the window size 3.77 s for the averaging. In ConceFT, the first 2 Hermit polynomials with the window size 3.77 s and 100 linear combinations are chosen: that is, *J *= *2* and *N *= *100*. The SPWVD, CWD, and scalogram are calculated by the widely used public code http://tftb.nongnu.org. We followed the suggested parameters and windows to produce the figures. The results are shown in Fig. 3. It is clear that while all TF analysis approaches capture the dynamics, the tvPS generated by ConceFT is sharper and cleaner. In SPWVD and CWD, the low frequency component seems to be interrupted in the middle, which might lead to misinterpretation. In the scalogram, it is not even clear if the low frequency component exists. This is caused by the affine transform nature of CWT—a large scale is associated with the low frequency region, which blurs the dynamic information.

The Matlab code of ConceFT and the simulated signal generator can be downloaded from https://hautiengwu.wordpress.com/code/ for purposes of reproducibility.

### Extra-long-term ECG signals

**Fig. 4:**
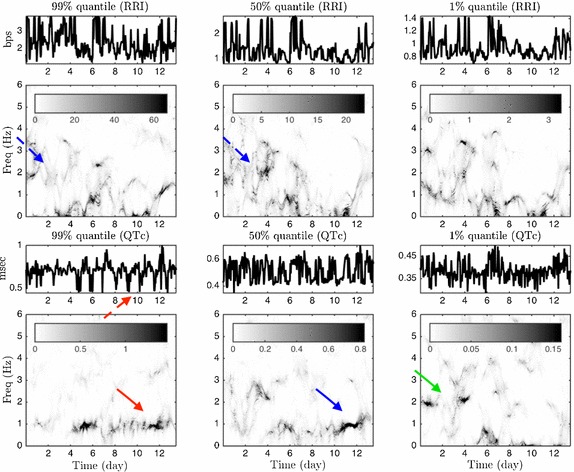
The ConceFT results of the first case. Top: the time-varying power spectrum (tvPS) of different time series related to the RRI; bottom: the tvPS of different time series related to the inverse of the QTc. The tvPS of the RRI is complicated. For *R*_*hour*,0.99_, *R*_*hour*,0.5_ and *R*_*hour*,0.01_, we could no visualize any dominant curve/line in their associated tvPS’s. However, a dominant blurred spectrum before day 6 ranging from 2 Hz to 4 Hz (indicated by the blue dashed arrow) indicates a roughly daily oscillation, but the oscillation is irregularly. For the QTc time series, the dark curve around 1 Hz after day 4 (indicated by the red arrow) in the tvPS of *Q*_*hour*,0.99_ indicates a daily oscillation of the QTc (indicated by the red dashed arrow). In the tvPS of *Q*_*hour*,0.5_, although we could still see a dark curve around 1 Hz after day 4, it is ``weakened’’ (indicated by the blue arrow) between day 4 and day 10, which indicates that although the signal *Q*_*hour*,0.5_ does have an daily oscillatory pattern, it is not as strong as the signal *Q*_*hour*,0.99_. We could not see a daily oscillation in *Q*_*hour*,0.01_, while there seems to have a half day oscillation before day 4 in *Q*_*hour*,0.01_ as we could see a curve at frequency 2 Hz before day 4 (indicated by the green arrow)

We used four long-term ECG recordings (323, 335, 333, and 306 h, respectively). To run ConceFT on the above-mentioned time series, we used *J *= *3* windows and *N *= *100* linear combinations to evaluate the tvPS of each considered time series. The analysis results of the first subject are shown in Fig. 4. In this figure, the tvPSs of different time series related to the QTc are shown on the top, and those of different time series related to the inverse of the RRI are shown on the bottom.

The tvPSs shown in Fig. 4 could be interpreted as follows: First, the dark curve around 1 Hz after day 4 (indicated by the red arrow) in the tvPS of *Q*_*hour*,0.99_ indicates a daily oscillation of the QTc. There is no obvious curve before day 4 in the tvPS of *Q*_*hour*,0.99_, which indicates that there is no obvious oscillatory pattern before day 4. On the other hand, the non-dominant concentrated curve but a blurred spectrum suggests that there are some oscillatory irregular patterns. For example, in Fig. 4, we could not visualize any dominant curve/line in the tvPS of *R*_*hour*,0.99_, but a dominant blurred spectrum before day 8 ranging from 0 to 4 Hz could be perceived. This indicates a roughly daily oscillation, but the oscillation is irregularly in the range of 0 to 4 Hz.

**Fig. 5:**
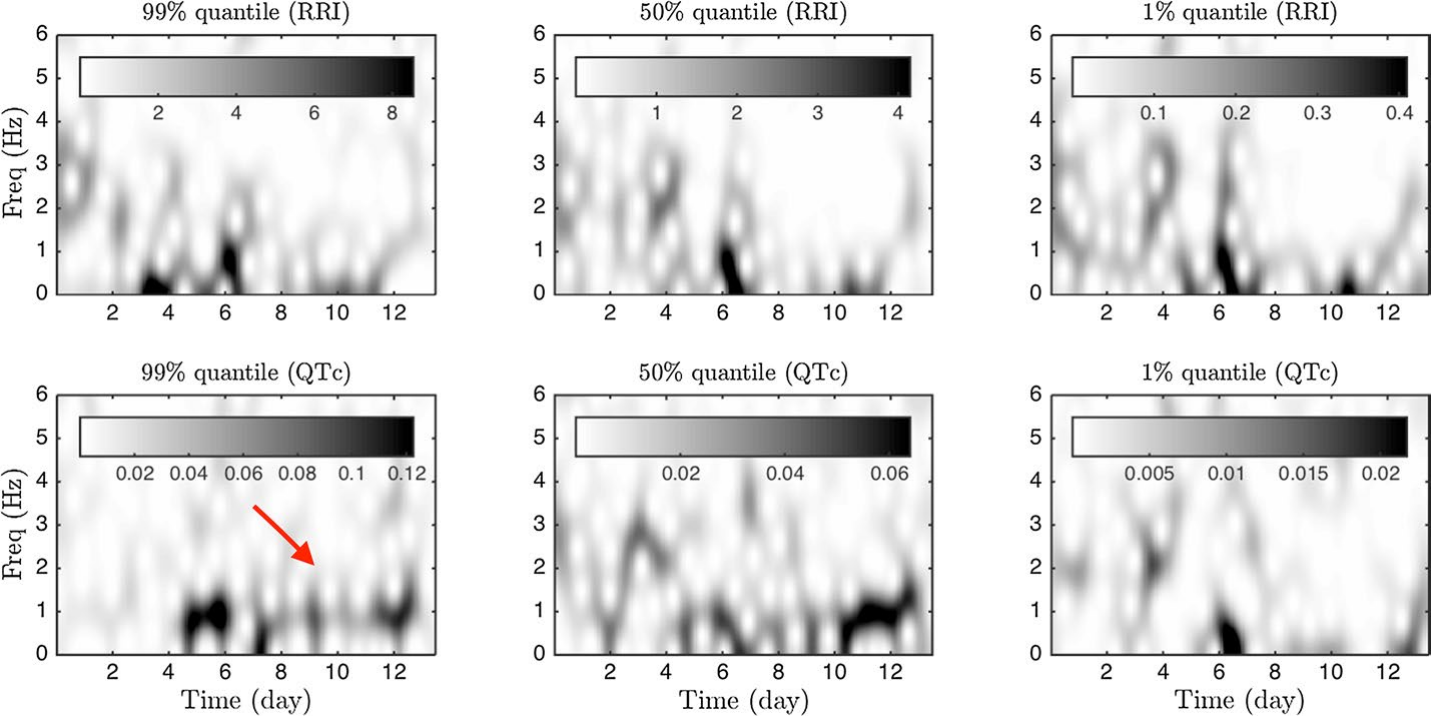
The spectrogram results of the first case for a comparison. Top: the spectrogram of different time series related to the RRI; bottom: the spectrogram of different time series related to the inverse of the QTc. Compared with the tvPS provided by ConceFT, the spectrogram is blurred and the pattern is less easy to identify. For example, the daily oscillation (indicated by the red arrow) after day 4 is very blurred

In Fig. 5, we show a comparison of the tvPS constructed from ConceFT and the spectrogram of time series considered in Fig. 4. Compared with Fig. 4, it is clear that overall the spectrogram is blurred and less easy to directly identify the 1 Hz oscillatory component in the *Q*_*hour*,0.99_ time series after day 4. This blurring comes from the uncertainty principle and it inevitable for all linear-type TF analysis methods. On the other hand, ConceFT takes the phase information of the signal and sharpens the TF representation, and hence the user could identify the possible information hidden inside the signal. This finding is consistent with the result shown in the simulated signal.

It is not surprising that tvPS’s of different time series have different behavior since they catch different aspects of of autonomic system. For example, *R*_*hour*,0.99_ provides partial information about the sympathetic tone, since in general we observe a fast heart rate when the sympathetic tone is active. Similarly, *R*_*hour*,0.01_ provides partial information about the parasympathetic tone. The results of the other three cases are shown in the Additional file 1.

## Discussion

In this report we presented a new approach for analysis of HRV and QT variability using data from long-term ECG recording. As proof of concept we applied this approach to four 2-week ECG recordings, and compared the results with other common TF approaches in a simulated signal. We showed that our proposed approach has the potential to enhance utilization of data collected from the current extra-long ECG recordings without the limitations of the currently applied TF analysis methods for 24–48 h recordings. Further research is needed to validate the HRV and QT variability indices obtained by our approach in terms of their association with outcomes.

From the machine learning perspective, summarizing the information in the tvPS by the NRR index is an unsupervised dimension reduction step. It certainly loses information, and the lost information might be critical for some applications. The NRR index was considered mainly to quantify the RR interval variability caused by the respiratory sinus arrhythmia under general anesthesiology [23]. For different applications, based on different physiological situations, we could define different indices in the future. Another approach is via the unsupervised learning approach to organize the tvPS, and/or other commonly considered HRV indices in the files [6]. By viewing ConceFT as a tool of unraveling dynamic information inside the RR interval or QT interval time series, we could further consider the idea of recurrent plot [38] or nonlocal pattern like phase-rectified signal averaging [44] to explore the signal, and apply it to study other topics, like the coupling between QT variability and HRV [45], and IHR estimated from modalities other than ECG [46–48]. We mention that after extracting features from either defining new indices or via the nonlinear dimensional reduction technique, we could apply any suitable supervised learning technique to connect the extracted information from the long-term ECG signal with the clinically interesting facts and establish a prediction model. We leave these opportunities to future work.

## Conclusions

With the emergence of ECG recordings that go several days and weeks, development of a new approach to measure HRV, particularly one that can capture the dynamics with sharp and stable instantaneous spectral content, is needed to utilize the full potential of data generated from such extra-long-term ECG recordings. We demonstrated a new approach to study HRV and QT variability in extra-long-term ECG recording using a modern time–frequency analysis tool, ConceFT. A validation with a larger database with clinical outcomes is needed and will be carried out in our future work.

## Additional file

**Additional file 1: Figure S1.** The ConceFT results of the second case. Top: the time-varying power spectrum (tvPS) of different time series related to the inverse of the RRI; bottom: the tvPS of different time series related to the QTc. The tvPS of the RRI shows an oscillatory pattern. For *R*_*hour*,0.99_, we found a 2 Hz oscillation around day 4 to day 9, which comes from the regular spikes indicated by the red arrows. Note that the curve around 4 Hz is the multiple of the 2 Hz oscillation due to the spiky shape of the oscillation. For* R*_*hour*,0.5_ and* R*_*hour*,0.01_, we could visualize a dominant line at 1 Hz after day 7, which is indicated by the blue arrow. Note that the oscillation could also be visualized in* R*_*hour*,0.5_ and* R*_*hour*,0.01_. The different behaviour of* R*_*hour*,0.99_,* R*_*hour*,0.5_ and* R*_*hour*,0.01_ comes from the fact that they capture different physiological information. The QTc time series has a more complicated structure. Actually, it is not easy to recognize any line/curve that lasts long enough, except the 1 Hz oscillation from day 7 to day 10 in* Q*_*hour*,0.5_ indicated by the green arrow. **Figure S2.** The spectrogram results of the second case for a comparison. **Figure S3.** The ConceFT results of the third case. Top: the time-varying power spectrum (tvPS) of different time series related to the inverse of the RRI bottom: the tvPS of different time series related to the QTc. The tvPS of the RRI shows an oscillatory pattern. For* R*_*hour*,0.99_, we found a 1 Hz oscillation from day 2 to day 8, which reflects the oscillatory pattern in the signal indicated by the red arrows. The line around 1 Hz gets stronger in* R*_*hour*,0.5_ and* R*_*hour*,0.01_, which is indicated by the blue and green arrows. The QTc time series has an oscillatory pattern that could be confirmed by the line around 1 Hz. However, the 1 Hz oscillation is not very stable in* Q*_*hour*,0.99_ and * Q*_*hour*,0.5_, since the line around 1 Hz is fluctuating. Compared with * Q*_*hour*,0.99_ and * Q*_*hour*,0.5_, *Q*_*hour*,0.01_ has a more stable 1 Hz oscillation, but only before day 13. **Figure S4.**The spectrogram results of the third case for a comparison. **Figure S5.** The ConceFT results of the fourth case. Top: the time-varying power spectrum (tvPS) of different time series related to the inverse of the RRI bottom: the tvPS of different time series related to the QTc. The tvPS of the RRI shows an oscillatory pattern. For* R*_*hour*,0.99_, we found a 2 Hz oscillation around day 5 to day 8 indicated by the red arrow, which come from the regular spikes indicated by the red dash arrow. For* R*_*hour*,0.01_, we could visualize a dominant line at 1 Hz before day 9 that fluctuates up to 1.5 Hz from day 9 to day 12. The curve indicated by the green arrow. The QTc time series has a more complicated structure and no line/curve could be recognized from the tvPS. **Figure S6.** The spectrogram results of the fourth case for a comparison.
