# Supplementary material for: A new approach for analysis of heart rate variability and QT variability in long-term ECG recording
Source: Biomed Eng Online. 2018 May 3;17:54. doi: 10.1186/s12938-018-0490-8 (PMC5932763; doi:10.1186/s12938-018-0490-8)
Supplement: Supplementary file 1 — Additional file 1: Figure S1. The ConceFT results of the second case. Top: the time-varying power spectrum (tvPS) of different time series related to the inverse of the RRI; bottom: the tvPS of different time series related to the QTc. The tvPS of the RRI shows an oscillatory pattern. For Rhour,0.99, we found a 2 Hz oscillation around day 4 to day 9, which comes from the regular spikes indicated by the red arrows. Note that the curve around 4 Hz is the multiple of the 2 Hz oscillation due to the spiky shape of the oscillation. For Rhour,0.5 and Rhour,0.01, we could visualize a dominant line at 1 Hz after day 7, which is indicated by the blue arrow. Note that the oscillation could also be visualized in Rhour,0.5 and Rhour,0.01. The different behaviour of Rhour,0.99, Rhour,0.5 and Rhour,0.01 comes from the fact that they capture different physiological information. The QTc time series has a more complicated structure. Actually, it is not easy to recognize any line/curve that lasts long enough, except the 1 Hz oscillation from day 7 to day 10 in Qhour,0.5 indicated by the green arrow. Figure S2. The spectrogram results of the second case for a comparison. Figure S3. The ConceFT results of the third case. Top: the time-varying power spectrum (tvPS) of different time series related to the inverse of the RRI bottom: the tvPS of different time series related to the QTc. The tvPS of the RRI shows an oscillatory pattern. For Rhour,0.99, we found a 1 Hz oscillation from day 2 to day 8, which reflects the oscillatory pattern in the signal indicated by the red arrows. The line around 1 Hz gets stronger in Rhour,0.5 and Rhour,0.01, which is indicated by the blue and green arrows. The QTc time series has an oscillatory pattern that could be confirmed by the line around 1 Hz. However, the 1 Hz oscillation is not very stable in Qhour,0.99 and Qhour,0.5, since the line around 1 Hz is fluctuating. Compared with Qhour,0.99 and Qhour,0.5, Qhour,0.01 has a more stable 1 Hz oscillation, [file 12938_2018_490_MOESM1_ESM.pdf]

Online Supplementary of
A new approach for analysis of heart rate variability and QT variability in long-term ECG recording

Hau-Tieng Wu, MD, PhD[1,2]; Elsayed Z. Soliman, MD, MSc, MS[3,4]

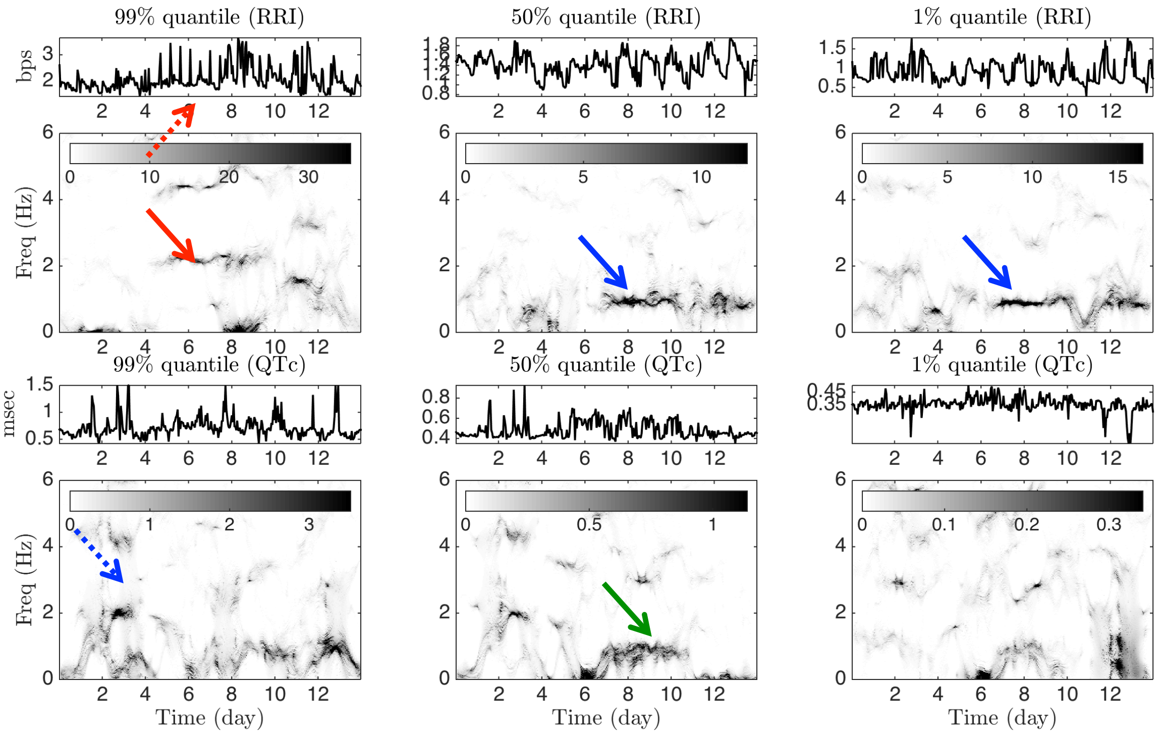

Figure S1. The ConceFT results of the second case. Top: the time-varying power spectrum (tvPS) of different time series related to the inverse of the RRI; bottom: the tvPS of different time series related to the QTc. The tvPS of the RRI shows an oscillatory pattern. For $R_{hour,0.99}$, we found a 2Hz oscillation around day 4 to day 9, which comes from the regular spikes indicated by the red arrows. Note that the curve around 4Hz is the multiple of the 2Hz oscillation due to the spiky shape of the oscillation. For $R_{hour,0.5}$ and $R_{hour,0.01}$, we could visualize a dominant line at 1Hz after day 7, which is indicated by the blue arrow. Note that the oscillation could also be visualized in $R_{hour,0.5}$ and $R_{hour,0.01}$. The different behavior of $R_{hour,0.99}$, $R_{hour,0.5}$ and $R_{hour,0.01}$ comes from the fact that they capture different physiological information. The QTc time series has a more complicated structure. Actually, it is not easy to recognize any line/curve that lasts long enough, except the 1Hz oscillation from day 7 to day 10 in $Q_{hour,0.5}$ indicated by the green arrow.

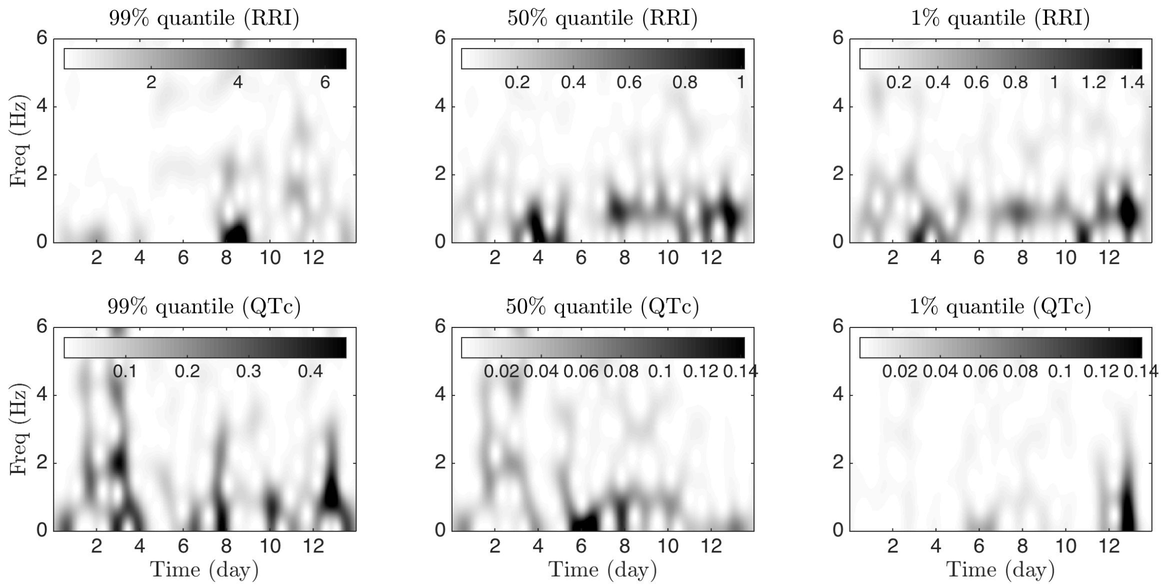

Figure S2. The spectrogram results of the second case for a comparison.

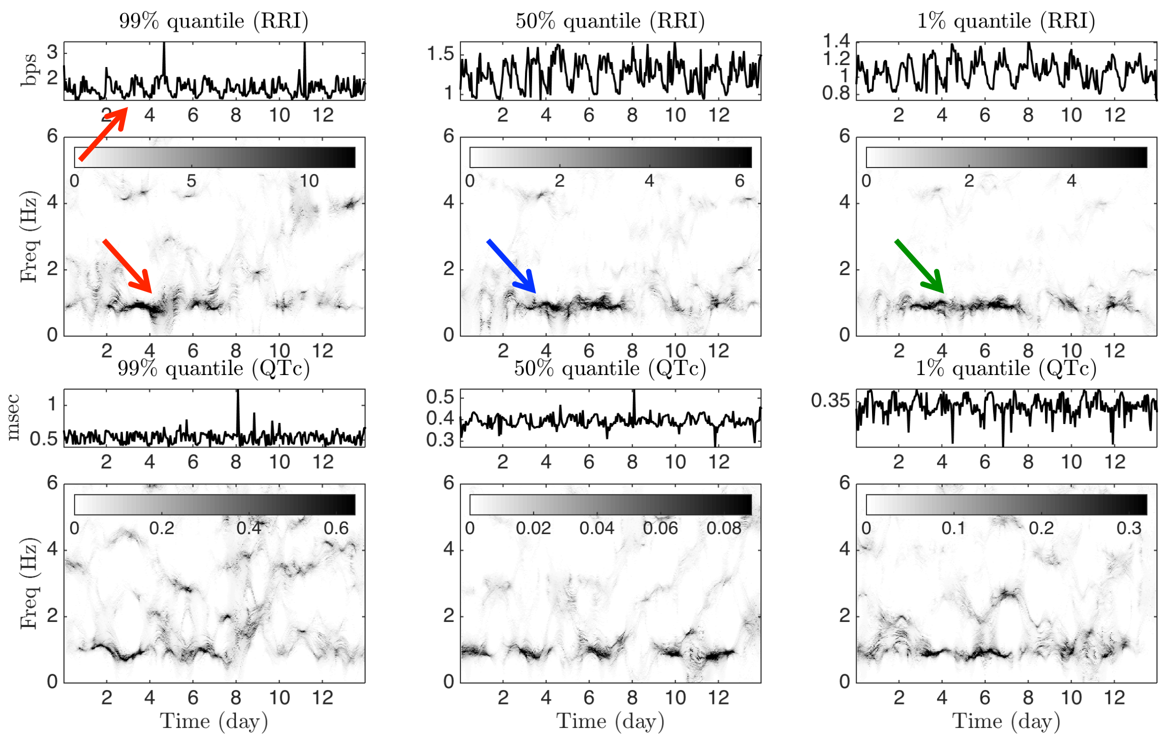

Figure S3. The ConceFT results of the third case. Top: the time-varying power spectrum (tvPS) of different time series related to the inverse of the RRI bottom: the tvPS of different time series related to the QTc. The tvPS of the RRI shows an oscillatory pattern. For $R_{hour,0.99}$, we found a 1Hz oscillation from day 2 to day 8, which reflects the oscillatory pattern in the signal indicated by the red arrows. The line around 1Hz gets stronger in $R_{hour,0.5}$ and $R_{hour,0.01}$, which is indicated by the blue and green arrows. The QTc time series has an oscillatory pattern that could be confirmed by the line around 1Hz. However, the 1Hz oscillation is not very stable in $Q_{hour,0.99}$ and $Q_{hour,0.5}$, since the line around 1Hz is fluctuating. Compared with $Q_{hour,0.99}$ and $Q_{hour,0.5}$, $Q_{hour,0.01}$ has a more stable 1Hz oscillation, but only before day 13.

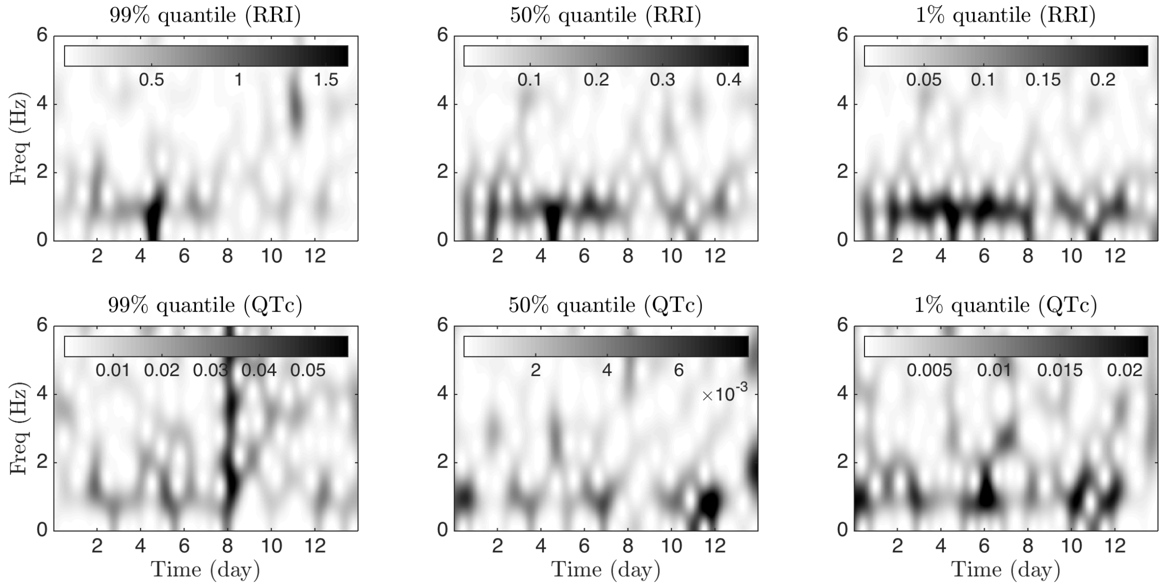

Figure S4. The spectrogram results of the third case for a comparison.

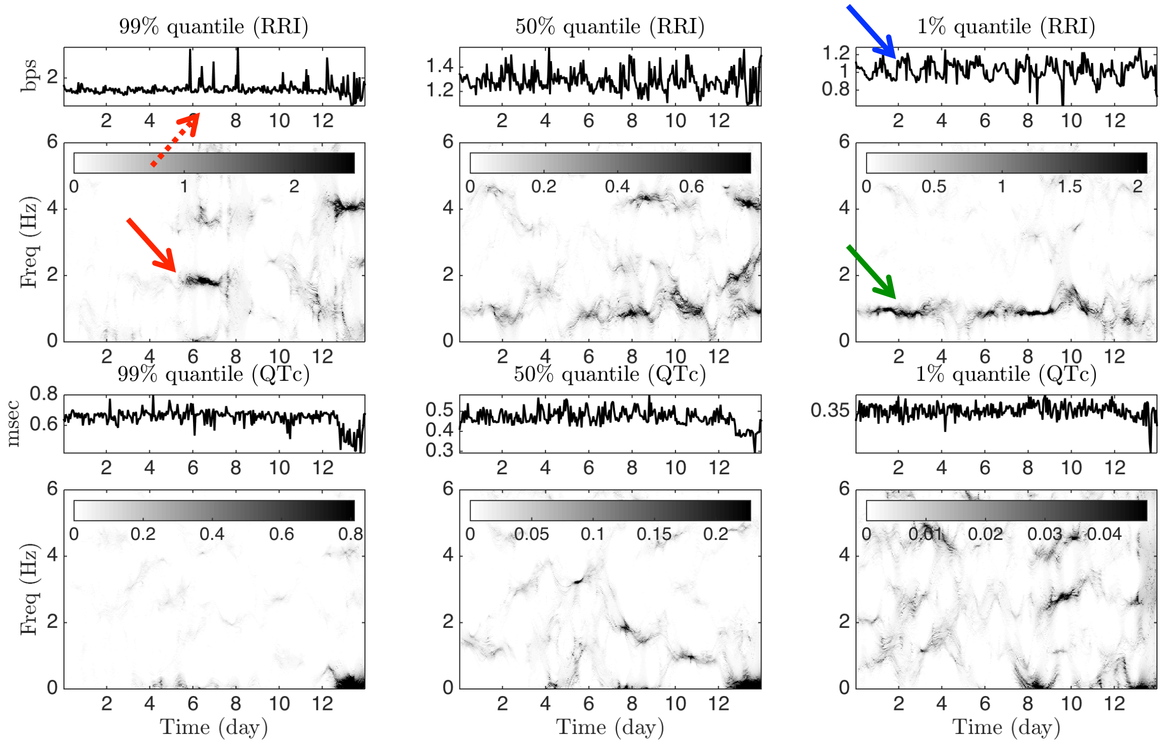

Figure S5. The ConceFT results of the fourth case. Top: the time-varying power spectrum (tvPS) of different time series related to the inverse of the RRI bottom: the tvPS of different time series related to the QTc. The tvPS of the RRI shows an oscillatory pattern. For $R_{hour,0.99}$, we found a 2Hz oscillation around day 5 to day 8 indicated by the red arrow, which come from the regular spikes indicated by the red dash arrow. For $R_{hour,0.01}$, we could visualize a dominant line at 1Hz before day 9 that fluctuates up to 1.5Hz from day 9 to day 12. The curve indicated by the green arrow. The QTc time series has a more complicated structure and no line/curve could be recognized from the tvPS.

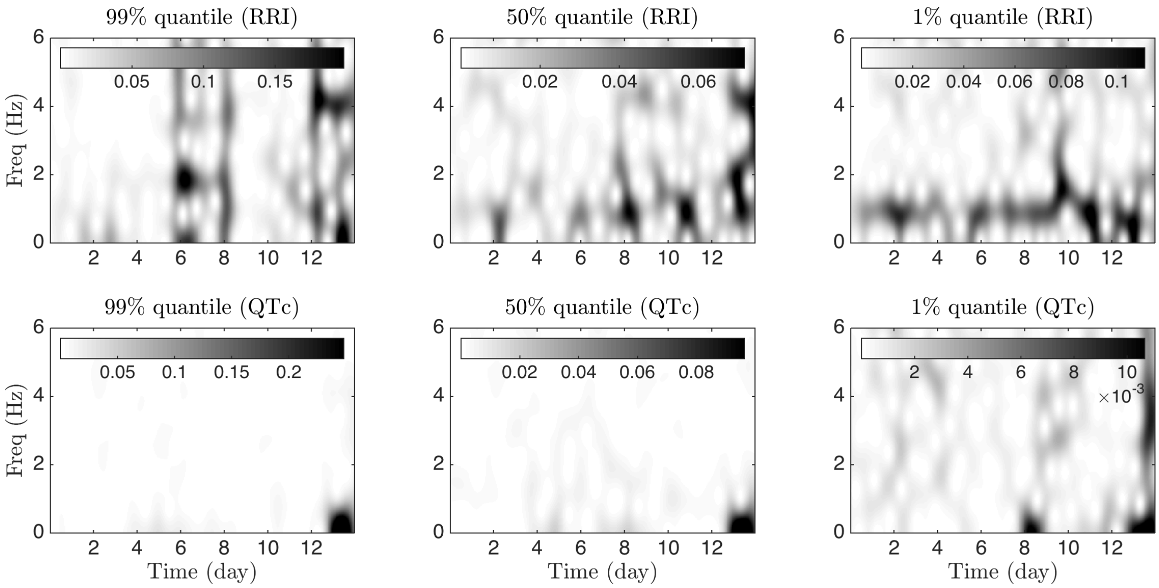

Figure S6. The spectrogram results of the fourth case for a comparison.
